# Multidimensional Civic Engagement in Later Life in 32 European Countries—an Exploration of the Roles of Socio-Structural Resources and Welfare State Commitment

**DOI:** 10.1177/01640275251351016

**Published:** 2025-06-11

**Authors:** Marina Näsman, Fredrica Nyqvist, Mikael Nygård, Toon Vercauteren, Sarah Dury, Rodrigo Serrat

**Affiliations:** 1 Faculty of Education and Welfare Studies, 1040 Åbo Akademi University, Vaasa, Finland; 2 Society and Ageing Research Lab (SARLab), 70493 Vrije Universiteit Brussel (VUB), Brussels, Belgium; 3 Department of Cognition, Development, and Educational Psychology, 16724 University of Barcelona, Barcelona, Spain

**Keywords:** multilevel analysis, political participation, volunteering, associational participation, informal caregiving

## Abstract

This study examines individual socio-structural resources and country-level welfare state commitment in relation to four different aspects of civic engagement in later life. Multilevel logistic regression was used to analyze data from the European Quality of Life Survey (EQLS) collected from people aged 65 and over in 32 European countries during 2016–2017 (*n* = 9265). On the individual level, socio-structural resources in terms of educational level, income, and self-rated health were positively related to formal volunteering, political participation, and associational participation. However, socio-structural resources seemed to be less important to informal caregiving. On the country-level, strong welfare state commitment, as measured by social expenditure, was positively associated with all four aspects of civic engagement. Cross-level interactions between socio-structural resources and welfare state commitment were statistically significant in part. The present study strengthens the view of civic engagement in later life as a multidimensional concept that is shaped by individual and contextual factors.

## Introduction

Civic engagement, such as volunteering and political participation, is a central part of participatory democracy and social inclusion in later life (Serrat et al., 2020; Walsh et al., 2017). It also supports active, successful, and healthy aging (e.g., Rowe & Kahn, 2015; WHO, 2020). Considering the globally increasing share of older adults (e.g., WHO, 2015), civic engagement is an important topic to pay attention to, as older adults constitute a major, sometimes untapped, social resource and a progressively powerful segment of the population in the political sphere (Serrat et al., 2020, 2022). Individual resources such as educational level, income, and health, which are also known as socio-structural resources, have often been associated with the possibilities for civic engagement (e.g., Schlozman et al., 2018; Serrat et al., 2023). However, research adopting a multidimensional approach to civic engagement in later life is scarce, highlighting the need to explore whether the same predictors influence different forms of civic engagement in a comparative manner. Moreover, to what extent does welfare state commitment play a role—that is, whether more extensive welfare state commitment stimulates or impedes civic engagement—remains unclear (cf. Esping-Andersen, 1990; Warburton & Jeppsson Grassman, 2011). In addition, the potential interaction of welfare state commitment and socio-structural resources must be addressed to understand their combined impact on civic engagement. Accordingly, this study examines socio-structural resources, welfare state commitment, and their interactions in relation to multidimensional civic engagement in later life in 32 European countries.

## Multidimensional Civic Engagement and the Present Study

Serrat et al. (2022) defined civic engagement in later life as “… unpaid, non-professional activities aimed at seeking improved benefits for others, the community, or wider society, or impacting on collective decision-making processes”. In line with this definition, they consider civic engagement to be a multidimensional concept comprising two main parts, volunteering and political participation, which can be further divided into sub-types. Volunteering can be both formal and informal—for example, volunteer work in organizations (formal), caregiving, and other types of altruistic helping behaviors (informal). Informal helping behaviors include thus providing (unpaid) care for someone inside or outside one’s household (Herd & Meyer, 2002; Nesteruk & Price, 2011) as well as other ways of helping and supporting others (Kruse & Schmitt, 2015). Similarly, political participation refers to both formal (institutionalized) and informal (non-institutionalized) activities. Formal political participation includes voting and participation in political organizations, whereas informal political participation includes grassroots activities such as signing petitions, demonstrating, and boycotting (Goerres, 2009; Serrat et al., 2022). Associational participation could be considered a central part of civic engagement in later life owing to its collective and community-engaging nature that often contains voluntary and/or political features (Ekman & Amnå, 2012; Putnam, 2000). In this study, we thus focus on four types of civic activities: formal volunteering, informal caregiving, political participation, and associational participation.

Despite the increase in the number of scientific publications on civic engagement among older adults, especially during the last two decades, some evident knowledge gaps remain to be addressed (Serrat et al., 2020). First, not all aspects of civic engagement in later life have received the same level of research attention. For example, whereas formal volunteering has been the focus in most previous research, far less attention has been given to political participation (Serrat et al., 2020). Scholars have also underscored the importance of broadening the scope of research on civic engagement in later life to include informal helping behaviors within social networks and community settings beyond formal institutions, in order to comprehensively capture the diverse contributions of older adults to societal well-being (Celdrán & Chakur Kiss, 2025; Martínez et al., 2011). Accordingly, informal caregiving, albeit contested, has been brought forward as an unrecognized type of civic engagement that should be acknowledged (Herd & Meyer, 2002; Nesteruk & Price, 2011). Second, a need for more studies including contextual factors on the macro level has been raised (Lu et al., 2021), especially using a cross-country approach within a European context (Serrat et al., 2020). Older adults’ civic engagement can be assumed to be shaped by the sociopolitical context in which engagement occurs, for example in terms of welfare regime type (Esping-Andersen, 1990; Goerres, 2009). In support of this notion, differences in the levels of engagement among older adults between European countries have been noted with regard to, for example, formal volunteering (Hank & Erlinghagen, 2010; Lee, 2024; Morawski et al., 2022) and political participation (Goerres, 2009; Nyqvist et al., 2024), indicating generally higher rates in northern and western Europe than in southern and eastern Europe.

Third, whether the same predictors apply to different types of civic activities remains unclear. To our knowledge, only a few studies have included more than one aspect of civic engagement in later life and examined their predictors in a comparative manner (e.g., Grünwald et al., 2021; Kim, 2020; Scharn et al., 2019; Strauss, 2021). These studies indicate the presence of both similarities and differences in the factors associated with different activities. However, only some of these studies have included country-level factors such as Gross domestic product (GDP) per capita and public spending on welfare (Avital, 2017; Hank, 2011; Strauss, 2021). Although a handful of studies have included associational participation and political participation (Avital, 2017; Boerio et al., 2023; Serrat et al., 2023) these forms of civic engagement have rarely been included in gerontological research.

Taken together, examining civic engagement in later life from a multidimensional perspective and by including both individual- and country-level predictors is crucial. Better understanding of the dynamics of these components not only illuminates the pattern of civic engagement in later life but also offers insights essential for informing policies and interventions aimed at promoting active and healthy aging. The next sub-sections elaborate on how socio-structural resources and welfare state commitment could be expected to shape multidimensional civic engagement in later life and outline the three hypotheses to be tested.

### Socio-Structural Resources

Previous research posits that an individual, regardless of age, needs to be equipped with sufficient resources to participate in civic activities (Engelman et al., 2022; Nygård & Jakobsson, 2013; van Ingen & van der Meer, 2011). In particular, socio-structural resources, including educational level, economic situation, and health have been shown to contribute to the understanding of the enablers and barriers of civic engagement in later life (Serrat et al., 2023) and will therefore be in focus in this study. Socio-structural resources are thought to provide the means and skills necessary to civically engage (Einolf & Chambré, 2011; Schlozman et al., 2018). According to Einolf and Chambré (2011) higher levels of socio-structural resources, such as higher educational level, could be reflected in a lower threshold to both engaging in different activities and being asked to engage.

The few studies that have simultaneously examined more than one aspect of civic engagement in later life, akin to the present study, have shown mixed findings concerning the role of socio-structural resources. Some studies have indicated that the connection of socio-structural resources with informal caregiving is less evident than that with associational participation and formal volunteering (e.g., Avital, 2017; Grünwald, et al., 2021; Kim, 2020). For example, whereas better self-rated health has been positively associated with formal volunteering, poorer self-rated health has been associated with giving informal care (Grünwald et al., 2021). In a similar vein, inconsistent results have been reported for informal caregiving and formal volunteering regarding the influence of mental health (Choi et al., 2007; Hank, 2011; Kim, 2020) and chronic diseases (Hank, 2011; Scharn et al., 2019). Informal caregiving may thus be driven by other factors—such as emotional ties and family obligations (Choi et al., 2007; Hermansen, 2016)—beyond socio-structural resources. The evidence, however, is elusive and varies between the studies depending on the outcome and socio-structural resources under study. Nevertheless, we can outline (two) different expectations for how socio-structural resources affect multidimensional civic engagement:

H1 Having more socio-structural resources is positively associated with civic engagement. However, the role of socio-structural resources could be expected to differ to some extent for informal caregiving.

### Welfare State Commitment

As previously mentioned, differences in the levels of civic engagement in later life between the European countries have been noted (e.g., Goerres, 2009; Hank & Erlinghagen, 2010; Lee, 2024; Quashie et al., 2022). One explanation for these differences could be related to welfare state commitment (Warburton & Jeppsson Grassman, 2011)—in other words, to what extent the state promotes well-being among its citizens. For example, the relatively high levels of civic engagement noted in the Scandinavian countries have been attributed to institutional factors and egalitarian policies that stimulate participation (Henriksen et al., 2019). Correspondingly, the crowding-in versus crowding-out hypotheses have been brought forward as possible explanations for the relations between welfare expenditure and the propensity to volunteer (Baer et al., 2016; Hank, 2011; Stadelmann-Steffen, 2011). The crowding-in hypothesis posits that countries with high social expenditures encourage civic engagement through various direct and indirect supportive measures such as financing community programs that encourage engagement or improving infrastructure. In contrast, the crowding-out hypothesis argues that welfare state effort impedes civic engagement by undermining interpersonal help and reciprocity. In other words, voluntary organizations and individual contributions could be considered less important in a society with high levels of social expenditure. Of these two, the crowding-in hypothesis has received more empirical support (Baer et al., 2016), although evidence pointing to a crowding-out effect has also been found (Stadelmann-Steffen, 2011). Thus, the theoretical discussion on welfare state commitment leads to the following hypothesis:

H2 Stronger welfare state commitment (as measured by social expenditure) is positively associated with civic engagement in later life.

### The Interaction of Socio-Structural Resources and Welfare State Commitment

Previous research has proposed that some country-level factors can mitigate or boost the effect of individual-level factors. According to Baer et al. (2016), increased welfare state expenditure can increase formal volunteering among those with low levels of income or education, whereas the link seems to be weaker among those with higher levels of education and income. In a similar vein, van Ingen and van der Meer (2011) proposed a resource approach to explain the role of the welfare state, where two main mechanisms are thought to stimulate participation in voluntary associations by reducing inequalities: (1) the redistribution of individual resources and (2) the offer of collective resources. They found that welfare state expenditure moderates participatory inequalities in voluntary organizations with regard to income, education, and gender. More specifically, with higher levels of redistribution and offer of collective resources, the likelihood of participation in organizations increased among those with lower educational level, lower income, and women. Regarding volunteering, Stadelmann-Steffen (2011) argued that crowding-in occurs among those with low income but not among the higher social classes. A recent systematic literature review by Schröder and Neumayr (2023) has brought forward a resource hypothesis, which suggests that more equal societies enable people, especially those with fewer resources, to be civically engaged. Nevertheless, these potential interactions must be examined from a multidimensional perspective, considering various forms of civic engagement, and with a focus on older adults. Thus, the third hypothesis that will be tested in the empirical section is:

H3 The associations between individual-level socio-structural resources and civic engagement in later life are weaker in countries with stronger welfare state commitment.

## Research Design

### Data and Sample

The data were derived from the fourth wave of the European Quality of Life Survey (EQLS) collected in 2016–2017 (Eurofound, 2018). EQLS was developed to complement measures of economic growth and focuses on different dimensions of quality of life, quality of society, and quality of public services. For the purpose of the present study, EQLS offered a comprehensive assessment of multidimensional civic engagement across welfare state contexts encompassing 33 European countries: Albania, Austria, Belgium, Bulgaria, Cyprus, Croatia, Czech Republic, Denmark, Estonia, Germany, Greece, Finland, France, Hungary, Ireland, Italy, Lithuania, Luxembourg, Latvia, Malta, Montenegro, the Netherlands, North Macedonia, Poland, Portugal, Romania, Serbia, Slovenia, Slovakia, Spain, Sweden, Turkey, and the United Kingdom. Albania was, however, excluded from this study owing to missing data regarding social expenditure (see the “Predictors” section below). Individuals aged 18 and over with no upper age limit were invited to participate through multi-stage, stratified, random sampling (Eurofound, 2018). The sample of the current study comprised 9265 individuals aged 65 and older from 32 countries. The number of individuals per country ranged from 98 (Montenegro) to 519 (Italy). The survey was conducted via computer-assisted personal interviewing (CAPI). More information regarding data collection, including ethical considerations, can be found in Eurofound’s (2018) technical and fieldwork report. The data is available free of charge for non-commercial purposes and stored at the UK Data Service (European Foundation for the Improvement of Living and Working Conditions, 2018).

### Measures

#### Civic Engagement

Four aspects of civic engagement were included in the analyses as dependent variables: formal volunteering, informal caregiving, political participation, and associational participation.

Formal volunteering was assessed through whether the respondent had done unpaid voluntary work for (a) community and social services organizations; (b) educational, cultural, sports, or professional associations; or (c) other voluntary organizations. A dichotomous variable was created where an individual was considered to have done formal volunteering if they had volunteered in any of the above-mentioned organizations during the last 12 months (=1).

Informal caregiving was assessed with two indicators: (a) how often the respondent had cared for disabled or infirm family members, neighbors, or friends under 75 years old or (b) aged 75 or over. This reflects a broad conceptualization of informal care, acknowledging that caregiving can be directed toward different groups within one’s social network both inside and outside one’s household (e.g., Celdrán & Chakur Kiss, 2025; Martínez et al., 2011). These indicators were combined in a dichotomized variable. Answering every day, several days a week, 1–3 times a week, or less often to any of the two was counted as providing informal care (=1), whereas answering never to both was counted as not providing informal care (=0).

Political participation was operationalized by combining several indicators of formal and informal character into a sum variable: (a) performed voluntary work for social movements or charities; (b) performed voluntary work for political parties or trade unions; (c) attended a meeting of a trade union, political party, or political action group; (d) attended a protest or demonstration; (e) signed a petition; (f) contacted a politician or public official; (g) commented on a political or social issue online; or (h) boycotted certain products. These indicators had an acceptable level of internal consistency with a Cronbach’s alpha of 0.7 (e.g., Forero, 2014). A dichotomous variable was created where an individual was considered to have participated politically if they confirmed their participation in any of these activities during the last 12 months (=1).

Associational participation was determined based on how frequently the respondent participates in the social activities of a club, a society, or an association. The variable was dichotomized such that individuals were considered to participate in an association (coded as 1) if they selected any of the following options: every day or almost every day, at least once a week, 1–3 times a month, or less often. Those who selected ‘never’ were coded as 0.

#### Predictors

Of the individual-level variables, the measures of socio-structural resources, which included educational level, income, and self-rated health, were of main interest. Educational level was determined based on the question: “What is the highest level of education you completed?” It was categorized into three levels based on the 2011 International Standard Classification of Education (ISCED): low secondary or lower (ISCED 0–2), upper secondary or post-secondary (ISCED 3–4), and tertiary level of education (ISCED 5–8). Income was determined as the household’s total net income per month as reported by the respondent. The variable used for the analysis represents income quartiles equivalized by country to allow for cross-national comparison and was generated by EQLS. Self-rated health was assessed based on the question: “In general, how is your health?” Very good and good were combined into one category (=1) and fair, bad, and very bad into the other (=0).

Drawing on previous research on civic engagement in later life (e.g., Avital, 2017; Grünwald et al., 2021; Lu et al., 2021; Serrat et al., 2023), the following control variables were also included: age, sex, employment status, and living with a partner. Age was used as a continuous variable; male was coded as 0 and female as 1. Employment status was divided into two categories: employed (including self-employed and those who receive retirement pension but are still employed) (=1) and other (retired, unemployed, unable to work, or a full-time homemaker) (=0). The variable living with a partner refers to whether the respondent had a partner in the same household (no = 0, yes = 1).

The countries’ level of social expenditure in 2016 was used as a country-level predictor and proxy for welfare state commitment. While acknowledging that this measure may oversimplify the complexity of welfare state commitment, it is a widely used and comparable indicator that captures the overall scope of state involvement in social protection across countries (c.f., Obinger, 2021). The data were derived from Eurostat and measured as the percentage of the GDP of each country. The measure included social benefits (due to, e.g., sickness, disability, unemployment, and old age), administration costs, and other expenditure (Eurostat, 2025a). More information on the measure can be found in the metadata of social protection (Eurostat, 2025b). Data were available for all countries included in the EQLS 2016 except Albania.

### Analytic Strategy

Information on civic engagement and socio-structural resources was extracted as percentages and means according to country and for the total sample for descriptive analysis. The data derived from EQLS were weighted using the WCalib_crossnational_total calculated by the Eurofound (2018, pp. 58–61) to account for relative country and population sizes.

Multilevel logistic regression analyses using the *melogit* command in STATA 17 were performed, where individuals (level 1) were nested into countries (level 2). The feasibility of using multilevel modeling was first tested by running empty models for each type of civic engagement and checking for the intra class correlation (ICC) coefficient. A value greater than .05 can be considered to indicate sufficient clustering at level 2 (Heck et al., 2014). The ICC coefficient was .17 for political participation, .16 for formal volunteering, .22 for associational participation, and .08 for informal caregiving, which supports the choice of analysis. To test the hypotheses of the study, three models for each type of civic engagement were tested. Model 1 included individual-level socio-structural resources while controlling for age, sex, employment status, and living with a partner. Potential multicollinearity was checked for with satisfactory results (VIF = 1.24). In Model 2, welfare state commitment, as measured by country-level social expenditure, was added. Finally, building upon Model 2, cross-level interactions between socio-structural resources and social expenditure were tested (see Table A1). Statistically significant (*p* < .05) cross-level interactions were interpreted and illustrated with the help of margins plots. The regression analyses were weighted by Wcalib (Eurofound, 2018, pp. 58–61). Missing values were negligible for all variables (in the range of 0.0%–0.6%) except for income quartiles, where 1491 individuals (16.1%) were non-respondents. The number of respondents included in the regression analysis therefore ranged between 7702 and 7734 for the different types of civic engagement. Descriptive non-response analyses showed that those with missing information regarding income reported to a larger extent that it was easy to make ends meet, and that their health was good, than those with information on income (*p* < .05). No statistically significant differences were noted between the groups regarding age, educational level, or gender distribution. This indicated that the participants were not missing at random. A robustness check with missing income included as one category in the income quartile measure (cf. Steckermeier & Delhey, 2019) generated, however, similar results as the results included in this article (Not shown here, available on demand).

## Results

Descriptive information on each civic engagement type, the socio-structural resources, and the social expenditure per country and in the total sample is presented in Table 1. The percentage of respondents engaging in formal volunteering was 25.4 and ranged between 5.4 and 47.1 for the countries. The overall percentage of respondents engaging in informal caregiving, political participation, and associational participation was 26.2 (10.0%–44.8%), 28.1 (5.1%–65.0%), and 40.2 (7.1%–76.2%), respectively. With regard to socio-structural resources, 17.0% (5.1%–38.2%) had completed the tertiary educational level, 20.4% (8.8%–29.5%) were placed in the highest income quartile, and 45.2% (12.2%–74.1%) rated their health as good. The mean percentage of GDP spent on social expenditure among the included countries was 22.9 and ranged between 12.8 and 34.3. Regarding the control variables (not shown in the table), the mean age of the total sample was 73.5 (SD = 6.6); 57.4% were female, 4.5% were employed, and 49.6% had a partner in the household.

**Table 1.** Descriptive Information Regarding Multidimensional Civic Engagement, Socio-Structural Resources, and Social Expenditure According to Country and the Total Sample (*n* = 9265).

Country	Formal volunteering, %	Informal caregiving, %	Political participation, %	Associational participation, %	Tertiary educational level %	Highest income quartile %	Good self-rated health %	Social expenditure, % of country GDP
North								
Denmark (*n* = 307)	**41.0** ^**2**^	**36.2** ^**6**^	**45.0** ^**3**^	**69.2** ^**2**^	**36.1** ^**3**^	8.8	**62.1** ^**4**^	**32.5** ^**2**^
Finland (*n* = 433)	**35.5** ^**6**^	**44.0** ^**2**^	**45.8** ^**2**^	**68.9** ^**3**^	**38.2** ^**1**^	**23.0** ^**7**^	48.7	**31.6** ^**3**^
Netherlands (*n* = 287)	**35.9** ^**5**^	**33.2** ^**8**^	**34.6** ^**9**^	**54.4** ^**8**^	**25.6** ^**7**^	**21.1** ^**10**^	**63.6** ^**2**^	**29.5** ^**6**^
Sweden (*n* = 428)	**47.1** ^**1**^	**37.6** ^**5**^	**65.0** ^**1**^	**76.2** ^**1**^	**35.5** ^**5**^	13.3	**59.8** ^**6**^	**29.4** ^**7**^
West								
Austria (*n* = 151)	**40.5** ^**3**^	19.4	**39.0** ^**6**^	**62.7** ^**5**^	**37.8** ^**2**^	**24.2** ^**5**^	**56.9** ^**8**^	**29.8** ^**4**^
Belgium (*n* = 277)	**35.1** ^**7**^	**42.6** ^**3**^	30.8	**44.7** ^**10**^	19.7	**21.9** ^**9**^	**53.9** ^**10**^	**29.2** ^**8**^
France (*n* = 277)	**31.5** ^**10**^	**44.8** ^**1**^	**37.7** ^**7**^	**45.0** ^**9**^	19.4	**29.5** ^**1**^	**56.8** ^**9**^	**34.3** ^**1**^
Germany (*n* = 350)	**35.1** ^**7**^	18.5	**41.4** ^**4**^	**64.8** ^**4**^	**24.7** ^**9**^	19.3	46.3	**29.6** ^**5**^
Ireland (*n* = 261)	**32.2** ^**9**^	25.9	28.3	**55.5** ^**6**^	18.9	18.9	**74.1** ^**1**^	16.2
Luxembourg (*n* = 189)	28.1	22.7	**32.4** ^**10**^	37.2	**25.1** ^**8**^	**28.6** ^**2**^	**60.2** ^**5**^	20.6
United Kingdom (*n* = 385)	29.0	26.6	**40.7** ^**5**^	**55.5** ^**6**^	**24.5** ^**10**^	18.6	**62.9** ^**3**^	25.9
South								
Italy (*n* = 519)	22.8	27.0	19.2	31.0	8.4	**24.8** ^**4**^	33.3	**29.2** ^**8**^
Greece (*n* = 281)	6.4	17.3	12.8	15.3	12.9	20.4	47.9	**26.5** ^**10**^
Malta (*n* = 328)	16.1	20.2	21.1	24.4	8.3	11.2	36.9	16.4
Portugal (*n* = 363)	14.6	13.1	9.6	20.0	7.0	17.2	23.1	25.1
Spain (*n* = 208)	12.0	20.2	8.6	27.7	10.0	17.2	48.2	23.8
East								
Bulgaria (*n* = 313)	7.4	12.3	7.2	9.0	19.0	13.0	30.6	17.3
Croatia (*n* = 212)	11.8	20.9	21.4	20.5	12.7	19.6	17.9	21.6
Cyprus (*n* = 336)	23.4	16.2	26.7	20.4	13.4	18.2	43.0	19.4
Czech Republic (*n* = 258)	15.0	22.5	21.9	33.2	11.7	9.4	34.8	18.7
Estonia (*n* = 331)	7.8	20.8	12.5	28.8	**26.2** ^**6**^	10.0	12.2	16.6
Hungary (*n* = 336)	7.4	10.9	9.8	18.2	10.5	17.4	23.5	18.8
Latvia (*n* = 367)	12.4	**28.6** ^**10**^	11.8	13.9	23.1	14.1	14.2	14.9
Lithuania (*n* = 342)	10.5	18.9	11.6	14.1	**36.0** ^**4**^	13.7	15.0	15.4
Montengero (*n* = 98)	10.9	**30.1** ^**9**^	15.3	18.2	5.5	20.3	20.3	18.7
North Macedonia (*n* = 241)	6.9	10.0	13.9	12.2	11.3	**25.0** ^**3**^	23.1	14.2
Poland (*n* = 239)	14.3	14.1	10.2	13.7	9.9	16.2	25.5	21.0
Romania (*n* = 290)	5.4	12.5	5.1	7.1	6.9	19.9	14.9	14.6
Serbia (*n* = 194)	12.8	**35.3** ^**7**^	17.6	17.0	5.5	**22.4** ^**8**^	19.5	20.3
Slovenia (*n* = 273)	21.5	19.1	17.4	41.0	12.6	**24.2** ^**5**^	36.7	23.2
Slovakia (*n* = 279)	10.8	18.2	7.9	31.3	11.2	13.0	22.8	18.4
Turkey (*n* = 112)	**36.2** ^**4**^	**38.2** ^**4**^	**36.5** ^**8**^	27.4	5.1	16.7	**57.3** ^**7**^	12.8
Total sample (*n* = 9265)	25.4	26.2	28.1	40.2	17.0	20.4	45.2	22.9

*Note.* The values of the top ten countries for each variable are in bold. The superscript following the value denotes the country’s ranking per column. The percentages are weighted (WCalib_crossnational_total).

H1 and H2 were tested using the two-level binary logistic regression models presented in Table 2. Model 1 contained socio-structural resources on the individual level, whereas Model 2 additionally included level of social expenditure on the country level. The results showed that having a higher educational level was associated with a higher likelihood of engaging in all types of civic engagement except informal caregiving. A clear gradient for educational level was noted for formal volunteering, political participation, and associational participation; the odds ratios were higher for tertiary education than for upper secondary or post-secondary education in all three cases. Higher income was positively associated with all types of civic engagement. However, the association was not statistically significant when comparing the lowest (first) and second quartile for formal volunteering and associational participation. In addition, the association between income and informal caregiving was statistically significant only for the third quartile compared with the lowest quartile. Finally, a statistically significant association of having good self-rated health was found with formal volunteering and associational participation but not with political participation and informal caregiving. All these associations remained in Model 2. Higher social expenditure was associated with a higher likelihood of engaging in all four types of civic engagement. In comparison with Model 1, the ICC values in Model 2 were lower when the country-level variable was added, indicating that some of the country-level variation could be explained by social expenditure.

**Table 2.** Two-Level Binary Logistic Regression Models Testing for the Association Between Socio-Structural Resources on Level 1, Social Expenditure on Level 2 and Each Type of Civic Engagement.

	Formal volunteering (*n* = 7716)		Informal caregiving (*n* = 7702)		Political participation (*n* = 7734)		Associational participation (*n* = 7707)	
	OR (95% CI)	*p*-value	OR (95% CI)	*p*-value	OR (95% CI)	*p*-value	OR (95% CI)	*p*-value
Model 1								
Educational level								
Low secondary or lower	1		1		1		1	
Upper secondary or post-secondary	1.59 (1.23–2.06)	<.001	1.11 (0.89–1.39)	.354	1.85 (1.53–2.22)	<.001	1.72 (1.39–2.14)	<.001
Tertiary	2.40 (1.81–3.18)	<.001	1.18 (0.89–1.55)	.249	3.69 (2.91–4.70)	<.001	2.55 (1.98–3.31)	<.001
Income quartile								
Lowest	1		1		1		1	
Second	1.20 (0.95–1.53)	.131	1.21 (0.99–1.47)	.057	1.27 (1.01–1.60)	.040	1.21 (0.97–1.50)	.087
Third	1.42 (1.05–1.93)	.022	1.37 (1.09–1.73)	.006	1.61 (1.28–2.02)	<.001	1.44 (1.21–1.85)	.004
Highest	1.68 (1.23–2.31)	.001	1.15 (0.89–1.48)	.282	1.72 (1.34–2.21)	<.001	1.59 (1.24–2.04)	<.001
Self-rated health								
Poor	1		1		1		1	
Good	1.24 (1.04–1.46)	.015	1.09 (0.95–1.25)	.223	1.21 (0.99–1.45)	.051	1.58 (1.32–1.89)	<.001
Constant	1.65 (0.40–6.90)	.492	2.32 (0.83–6.49)	.109	2.52 (0.78–8.15)	.124	1.21 (0.49–2.97)	.673
Intra class correlation	.1541076		.0830088		.1805941		.20278	
AIC	5924.064		6726.375		5983.468		6826.125	
BIC	6007.477		6809.766		6066.909		6909.524	
Log-pseudolikelihood	−2950.0322		−3351.188		−2979.734		−3401.0625	
Model 2								
Educational level								
Low secondary or lower	1		1		1		1	
Upper secondary or post-secondary	1.59 (1.22–2.06)	.001	1.11 (0.88–1.40)	.352	1.84 (1.52–2.23)	<.001	1.72 (1.39–2.14)	<.001
Tertiary	2.38 (1.79–3.17)	<.001	1.17 (0.88–1.55)	.271	3.68 (2.88–4.71)	<.001	2.56 (1.97–3.32)	<.001
Income quartile								
Lowest	1		1		1		1	
Second	1.20 (0.95–1.52)	.134	1.21 (0.99–1.47)	.058	1.27 (1.01–1.60)	.043	1.21 (0.97–1.50)	.090
Third	1.42 (1.05–1.92)	.022	1.37 (1.09–1.72)	.006	1.60 (1.28–2.01)	<.001	1.43 (1.12–1.84)	.005
Highest	1.68 (1.22–2.30)	.001	1.15 (0.89–1.47)	.287	1.71 (1.33–2.20)	<.001	1.58 (1.23–2.03)	<.001
Self-rated health								
Poor	1		1		1		1	
Good	1.23 (1.04–1.46)	.017	1.08 (0.94–1.23)	.274	1.20 (0.99–1.45)	.054	1.58 (1.32–1.88)	<.001
Social expenditure	1.08 (1.03–1.12)	<.001	1.04 (1.01–1.08)	.007	1.09 (1.04–1.13)	<.001	1.11 (1.08–1.16)	<.001
Constant	0.32 (0.04–2.32)	.257	0.87 (0.25–2.97)	.820	0.40 (0.06–2.54)	.330	0.11 (0.03–0.46)	.002
Intra class correlation	.1051813		.0634448		.1227197		.1095577	
AIC	5913.039		6719.825		5971.87		6806.087	
BIC	6003.402		6810.165		6062.264		6896.436	
Log pseudolikelihood	−2943.519		−3346.9125		−2972.9351		−3390.0435	

*Note.* Model 1 included socio-structural resources while Model 2 additionally included social expenditure. All models were adjusted for age, gender, employment status, and relationship status. The weight WCalib was applied to the regression analysis.

OR = Odds ratio, CI = Confidence interval, AIC = Akaike information criterion, BIC = Bayesian information criterion.

**Figure 1.**
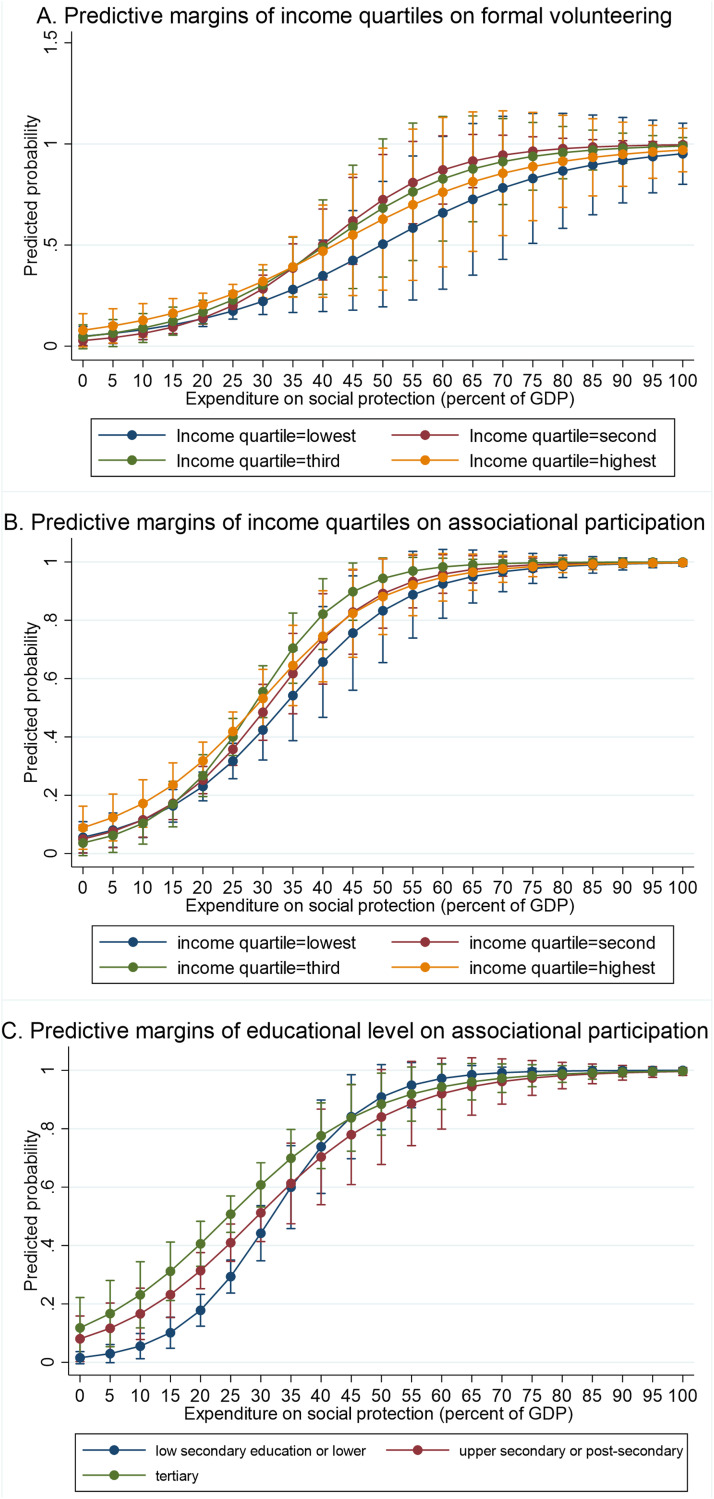
Cross-level interactions with 95% confidence intervals, given in predicted probabilities. *Note.* A. Associations between income quartiles and social expenditure on formal volunteering, B. Associations between income quartiles and social expenditure on associational participation, C. Associations between educational levels and social expenditure on associational participation.

Cross-level interactions between the socio-structural resources and social expenditure were tested to evaluate H3 (see Table A1). The results show statistically significant interactions of income and social expenditure on formal volunteering and of income and social expenditure as well as educational level and social expenditure on associational participation. The statistically significant interactions are presented as margins plots in Figure 1. The plots indicate that the roles of educational level and income are somewhat mitigated as the level of social expenditure increases—in other words, the differences in odds ratios between individuals with different educational levels and incomes are smaller when the country spends a larger share of the GDP on social expenditure. However, the interaction between income and social expenditure was statistically significant only between the lowest and second quartile for formal volunteering and between the lowest and third quartile for associational participation. Likewise, the interaction of educational level and social expenditure was statistically significant only between the lowest (low secondary or lower) and the second (upper secondary or post-secondary) level of education for associational participation.

## Discussion

The present study examined the variations and predictors of civic engagement in later life across 32 European countries by including formal volunteering, informal caregiving, political participation, and associational participation. Our results suggest that in addition to the notable differences in socio-structural resources and welfare state commitment across countries, large variations in multidimensional civic engagement in later life exist across Europe. In line with previous research on civic engagement in later life (Goerres, 2009; Lee, 2024; Morawski et al., 2022), we noted higher rates of engagement in northern and western European countries.

Further, we found support for socio-structural resources being positively associated with civic engagement and for the differences in the predictors of informal caregiving (H1). Stronger welfare state commitment as measured by social expenditure was also positively related to all four types of civic engagement (H2). A mitigating role of welfare state commitment on the associations between socio-structural resources and civic engagement (H3) was only found in some instances—namely, the interaction between educational level and social expenditure on associational participation and that between income and social expenditure on associational participation and on formal volunteering.

Consistent with previous research (e.g., Avital, 2017; Kim, 2020; Nygård & Jakobsson, 2013), socio-structural resources were confirmed to be of importance to political participation, formal volunteering, and associational participation but less so for informal caregiving. Neither educational level nor self-rated health were associated with informal caregiving, and a clear relationship between income and informal caregiving could not be identified. One possible explanation could be that informal helping behavior is driven by other factors such as norms, values, necessity, and family ties (Choi et al., 2007; Hermansen, 2016) rather than by socio-structural resources. Furthermore, lower socio-structural resources can be associated with a higher propensity to provide informal care (Kim, 2020), although providing informal care could also result in reduced resources, such as losses in health (Bom et al., 2019). Longitudinal analyses would have been required to delve deeper into these associations.

Informal caregiving displayed less country level variation compared to the other dimensions of civic engagement and was also commonly reported in countries with strong welfare state commitment in our study (see Table 1) although a strong public sector could be assumed to reduce the need for informal care (e.g., Zigante, 2018, p. 14). Similarly, higher social expenditure was associated with a higher likelihood of informal caregiving (Table 2). It is possible that the ability to combine formal and informal care is higher in countries with stronger welfare commitment (Furfaro et al., 2024; Jegermalm & Fladmoe, 2019; Verbakel, 2018), meaning that caregiver burden could be higher in countries with less state support. In other words, welfare state commitment reflected in, for example, more generous formal care provision could encourage individuals to provide informal care as a complement to other care services (Verbakel, 2018). Variations in cultural values across the European countries, such as degree of familialism could also play a role in shaping informal care provision (Furfaro et al., 2024) At the same time, it could reflect an ongoing decrement of welfare state commitment, seen for example in the northern European countries, calling for an increased need for informal caregiving (Hermansen, 2016; Jegermalm & Fladmoe, 2019). Note, however, that we did not measure the intensity of informal caregiving in our study. Nevertheless, our results can work as a steppingstone into more in-depth studies on the role and nature of informal caregiving as a form of civic engagement in later life across countries and welfare state systems. Furthermore, given that informal caregiving plays a prominent role in European long-term care systems and social policies (Zigante, 2018), our results reiterate the importance of critically discussing informal caregiving in later life in the civic engagement debate, especially in terms of the potential benefits and harms of this type of engagement.

Our overall results validate H2 and lend support to the crowding-in hypothesis (cf. Baer et al., 2016; Hank, 2011): welfare state commitment seems to stimulate multidimensional civic engagement in later life. In other words, national incentives have the potential to increase the level of civic engagement and thereby promote participatory democracy and social inclusion of older people. While a previous study on older adults (Hank, 2011) have found corresponding results regarding volunteering and informal helping behaviors, our results go beyond these findings to concern also political participation, and associational participation. However, we found only some support for H3—namely that the role of socio-structural resources would be smaller in countries with stronger welfare state commitment. On the one hand, our analysis implies that welfare state commitment is boosting older adults’ civic engagement regardless of socio-structural resources. On the other hand, it indicates that having more socio-structural resources in later life is positively associated with civic engagement regardless of the welfare state context. Making civic activities accessible for all older adults, irrespective of educational level, income, and health is thus a challenge concerning all included countries.

The weak support for H3 may partly be explained by the fact that factors beyond resources, such as societal norms, social integration/connectedness, and power imbalances between different socio-economic groups, can interact with and shape the associations between individual-level factors and welfare state commitment, suggesting more complex underlying dynamics (cf. Dury et al., 2020; Schröder & Neumayr, 2023; Strauss, 2021). From a statistical perspective, a larger number of countries included in the analysis could also have enhanced statistical power, thereby facilitating the detection of statistically significant effects when introducing cross-level interactions. Additional studies on the relationship between individual-level factors and welfare state commitment are thus warranted. Nevertheless, our results suggest that welfare state commitment can mitigate the roles of educational level and income in relation to associational participation and on the role of income in relation to formal volunteering in later life. Taking the lens of a resource approach, as suggested by van Ingen and van der Meer (2011), the redistribution and provision of collective resources seem to be able to promote associational participation and formal volunteering for those with lower levels of education and income, thereby potentially contributing to the reduction of inequality in civic engagement in later life.

### Limitations

A multidimensional approach to studying civic engagement can contribute to a richer evidence base. The EQLS, albeit collected in 2016–2017, offered the most comprehensive coverage of civic engagement indicators available in existing cross-national European surveys. Nevertheless, how the civic activities were assessed could restrict the generalizability of the results. For example, informal helping behaviors, including both informal caregiving and other types of help, can be assessed in different ways (Herd & Meyer, 2002; Kruse & Schmitt, 2015), which could affect the possibility of comparing results across studies. In addition, no information was available on whether informal care was given within or outside one’s household (cf. Strauss, 2021) and it was not possible to distinguish whether the care was provided to kin or non-kin (cf. Quashie et al., 2022). Further, owing to the low number of older adults who stated their formal political participation in some countries in the data set, we were not able to separate formal and informal political participation in the analyses. The variables used in this study were all dichotomous measures of civic engagement, as the data set did not include information on intensity/frequency for all items. Thus, we cannot, for instance, rule out that considering the intensity of the activities engaged in would have given a more nuanced picture (cf. Bom et al., 2019; Morawski et al., 2022; Quashie et al., 2022). While the choice to focus on socio-structural resources was well-anchored in the civic engagement literature (Schlozman et al., 2018; Serrat et al., 2023) and related to availability in the data set, there are also other factors on the individual level, such as personality (cf. Gerber et al., 2010), that could contribute to the understanding of civic engagement in later life. Furthermore, additional drivers of civic engagement could also be expected to differ across dimensions but were not explored in the present study for consistency reasons. Conclusions regarding country-level differences in civic engagement rates should overall be drawn with caution. Other variables on the country level besides social expenditure, such as socio-cultural values and historical context, could also be expected to affect civic engagement (cf. Hank, 2011; Morawski et al., 2022; Strauss, 2021) but were excluded owing to, among other things, the relatively low number of clusters on level 2 (i.e., countries) which restricts the analytical design (Bryan & Jenkins, 2016). Nevertheless, the ICC was considerably lower in the regression analyses after introducing social expenditure, suggesting that this measurement explained some of the variance between the countries. Finally, considering the cross-sectional nature of the EQLS data, the analyses do not allow for drawing any conclusions regarding causality. It is for example plausible that there is a reciprocal relationship between civic engagement and socio-structural resources, where more resources can lead to more engagement but also vice versa. Longitudinal, or even life course, studies of multidimensional civic engagement could potentially bring some insights into these dynamics.

## Conclusion

The present study makes three main contributions to the existing literature on civic engagement in later life. First, considerable diversity exists in terms of civic engagement among older adults in different countries, and this needs to be considered when studying this phenomenon. Second, the multidimensionality of civic engagement must be acknowledged in both research and policy development, as reflected by, for example, the deviation in associations between socio-structural resources and informal caregiving compared with other forms of civic engagement. The study also sheds light on the dimensions that have been less studied in the gerontological literature, such as political participation. Third, welfare state commitment seems to boost civic engagement among older adults. From a policy perspective, the findings suggest that social expenditure pays off in terms of a more civically engaged older population. In turn, this increased engagement supports the welfare system through the active contributions of older individuals, creating a reciprocal feedback loop. Taken together, the results indicate that both investments in individual resources and broader societal support can play a key role in promoting civic engagement in later life. This highlights for instance the important role of social policy interventions on national level ensuring that basic needs are met as well as financial structures that enable organizations on the grassroot level to promote engagement.

## Data Availability

Data from the European Quality of Life Survey are stored with the UK Data Service and are available free of charge for non-commercial purposes.*

## Appendix

**Table A1.** Two-Level Binary Logistic Regression Models Testing for Cross-Level Interactions Between Socio-Structural Resources and Social Protection on Each Type of Civic Engagement.

	Formal volunteering (*n* = 7716)		Informal caregiving (*n* = 7702)		Political participation (*n* = 7734)		Associational participation (*n* = 7707)	
	OR (95% CI)	*p*-value	OR (95% CI)	*p*-value	OR (95% CI)	*p*-value	OR (95% CI)	*p*-value
Educational level								
Low secondary or lower	1		1		1		1	
Upper secondary or post-secondary	3.56 (1.10–11.54)	.034	1.30 (0.56–3.03)	.547	1.93 (0.84–4.44)	.121	5.56 (2.22–13.92)	<.001
Tertiary	5.36 (0.98–29.34)	.053	0.67 (0.19–2.41)	.542	4.44 (0.98–20.2)	.053	8.57 (2.20–33.43)	.002
Income quartile								
Lowest	1		1		1		1	
Second	0.55 (0.27–1.10)	.092	1.94 (1.04–3.62)	.037	1.14 (0.44–2.90)	.791	0.88 (0.41–1.89)	.744
Third	0.94 (0.33–2.68)	.915	1.96 (0.87–4.42)	.105	1.38 (0.68–2.82)	.372	0.63 (0.28–1.43)	.267
Highest	1.70 (0.58–4.93)	.331	0.99 (0.94–1.03)	.592	2.11 (0.97–4.57)	.059	1.69 (0.79–3.65)	.178
Self-rated health								
Poor	1		1		1		1	
Good	1.41 (0.69–2.86)	.340	1.02 (0.68–1.54)	.926	0.82 (0.39–1.70)	.593	1.49 (0.85–2.63)	.160
Social expenditure	1.09 (1.02–1.16)	.011	1.06 (1.01–1.10)	.008	1.08 (1.01–1.16)	.036	1.13 (1.07–1.19)	<.001
Educational level*Social expenditure								
Low secondary or lower	1		1		1		1	
Upper secondary or post-secondary	0.97 (0.93–1.01)	.154	0.99 (0.96–1.02)	.663	1.00 (0.97–1.03)	.912	0.95 (0.92–0.99)	.007
Tertiary	0.97 (0.91–1.03)	.310	1.02 (0.97–1.07)	.374	0.99 (0.94–1.05)	.793	0.95 (0.91–1.00)	.053
Income quartile*Social protection								
Lowest	1		1		1		1	
Second	1.03 (1.00–1.06)	.043	0.98 (0.95–1.01)	.153	1.00 (0.97–1.04)	.809	1.01 (0.98–1.05)	.456
Third	1.02 (0.97–1.06)	.460	0.98 (0.95–1.02)	.372	1.01 (0.98–1.04)	.704	1.03 (1.00–1.07)	.043
Highest	1.00 (0.95–1.05)	.991	0.99 (0.95–1.03)	.592	0.99 (0.96–1.02)	.603	1.00 (0.97–1.03)	.881
Self-rated health*Social protection								
Poor	1		1		1		1	
Good	0.99 (0.97–1.02)	.656	1.00 (0.98–1.02)	.800	1.02 (0.99–1.04)	.278	1.00 (0.98–1.03)	.878
Constant	0.25 (0.20–3.23)	.290	0.69 (0.18–2.67)	.593	0.44 (0.04–5.28)	.519	0.08 (0.01–0.46)	.005
Intra class correlation	.1136413		.0641406		.1263388		.1217706	
AIC	5910.328		6725.097		5980.475		6790.872	
BIC	6042.398		6857.132		6112.589		6922.92	
Log pseudolikelihood	−2936.164		−3343.548		−2971.237		−3376.436	

*Note.* The analyses were adjusted for age, gender, employment status, and relationship status. The weight WCalib was applied to the regression analysis.

OR = Odds ratio, CI = Confidence interval, AIC = Akaike information criterion, BIC = Bayesian information criterion.
